# Evaluation of Potential In Vitro Recombination Events in Codon Deoptimized FMDV Strains

**DOI:** 10.3390/v15030670

**Published:** 2023-03-02

**Authors:** Edward Spinard, Ian Fish, Paul A. Azzinaro, Monica Rodriguez-Calzada, Ethan J. Hartwig, George R. Smoliga, Aishwarya Mogulothu, Jonathan Arzt, Teresa de los Santos, Gisselle N. Medina

**Affiliations:** 1 Plum Island Animal Disease Center (PIADC), ARS, USDA, P.O. Box 848, Greenport, NY 11944, USA; 2 College of Veterinary Medicine, Kansas State University, Manhattan, KS 66506, USA; 3 ORISE-PIADC Research Participation Program, Oak Ridge, TN 37831, USA; 4 Department of Pathobiology and Veterinary Science, University of Connecticut, Storrs, CT 06269, USA; 5 National Bio and Agro-Defense Facility (NBAF), ARS, USDA, Manhattan, KS 66502, USA

**Keywords:** foot-and-mouth disease, FMDV, recombination, codon deoptimization

## Abstract

Codon deoptimization (CD) has been recently used as a possible strategy to derive foot-and-mouth disease (FMD) live-attenuated vaccine (LAV) candidates containing DIVA markers. However, reversion to virulence, or loss of DIVA, from possible recombination with wild-type (WT) strains has yet to be analyzed. An in vitro assay was developed to quantitate the levels of recombination between WT and a prospective A24-P2P3 partially deoptimized LAV candidate. By using two genetically engineered non-infectious RNA templates, we demonstrate that recombination can occur within non-deoptimized viral genomic regions (i.e., 3′end of P3 region). The sequencing of single plaque recombinants revealed a variety of genome compositions, including full-length WT sequences at the consensus level and deoptimized sequences at the sub-consensus/consensus level within the 3′end of the P3 region. Notably, after further passage, two recombinants that contained deoptimized sequences evolved to WT. Overall, recombinants featuring large stretches of CD or DIVA markers were less fit than WT viruses. Our results indicate that the developed assay is a powerful tool to evaluate the recombination of FMDV genomes in vitro and should contribute to the improved design of FMDV codon deoptimized LAV candidates.

## 1. Introduction

Foot-and-mouth disease (FMD) is a highly contagious disease of cloven-hoofed animals, including cows, pigs, sheep, and goats that is characterized by fever, lameness, and the formation of blisters on the toes, tongue, and utters of infected animals [1,2]. The causative agent of FMD, FMD virus (FMDV), belongs to the genus *Aphthovirus* of the *Picornaviridae* family. 

The genome of FMDV is an ~8.2 kb single-stranded, positive sense RNA molecule that encodes a single open-reading frame (ORF) [3,4]. The ORF is composed of four coding regions: Leader (L^pro^), P1 capsid coding region (VP4, VP2, VP3, and VP1), P2 non-structural coding region (2A, 2B, and 2C), and P3 non-structural coding region (3A, 3B1-3, 3C, and 3D) [3,4]. The ORF is flanked by the highly structured 5′ and 3′ untranslated regions [3,4]. L^pro^, 2A, and 3C process the polyprotein into mature proteins [4]. The genome is replicated by the RNA-dependent RNA polymerase (RdRp) encoded by 3D [4]. In short, a complex composed of RdRp and other viral proteins synthesize a negative-strand intermediate that serves as a template for positive-strand synthesis [4,5].

Although mortality is low, outbreaks of FMD may cause severe economic losses in endemic regions. FMD is estimated to account for a yearly loss of between US $6.5 and $21 billion worldwide [6]. Control strategies in FMD endemic areas include the use of vaccination with an adjuvanted chemically inactivated FMDV preparation; however, use of these vaccines is marked by several difficulties: (1) production requires a specialized high containment biosafety level III facility to limit risk of virus release, (2) a chain of cold storage is required to prevent spoilage, and (3) multiple injections are required due to the short-lived vaccine-induced immunity (reviewed in [7]). Current vaccine improvement efforts focus on addressing these challenges and, among others, include the development of novel vaccine platforms such as live attenuated vaccines (LAVs). In general, LAVs induce more rapid and long-term protection [8]. Optimally, vaccines should also contain DIVA (Differentiating Infected from Vaccinated Animals) markers to allow for the immunological differentiation between naturally infected and previously vaccinated animals [7,9]. 

Potential LAV strains of FMDV have been created by deleting regions or incorporating mutations that directly target the coding and non-coding regions of the genome. For instance, the deletion of L^pro^ (leaderless, LLV) or specific mutations in the L^pro^ SAP domain rendered attenuated viruses in vitro and in vivo [10,11,12]. However, these strategies have proved insufficient, as inoculation with the live LLV vaccine did not induce a protective neutralizing antibody response in swine or cattle, which was presumably due to excessive attenuation. Additionally, the attenuation of the FMDV L^pro^ SAP mutant only relied on two amino acid substitutions, thus posing a significant risk of reversion given the known high error rate of the FMDV RdRp [13]. Therefore, improved FMDV LAV candidates must contain the right level of attenuation to offer protection and an increased number of attenuating mutations to reduce the chances of reversion to virulence.

Recently, the creation of attenuated FMDV strains has shifted from the direct targeting of virulence factors to altering the codon bias (CB) or codon-pair bias (CPB) of the genome [14,15]. In short, all organisms exhibit a preference toward the use of certain codons or separately, codon pairs. Modifying the CB or CPB can lead to mutants harboring hundreds of synonymous mutations. Complete or partial genome codon deoptimization (CD) or codon-pair deoptimization (CPD) has been leveraged to create a multitude of attenuated viruses [16,17,18,19,20,21,22]. Indeed, CPD of the FMDV P1 capsid coding region of strain A12 and CD of the P2 and/or P3 non-structural (NS) coding region of FMDV A24 has led to attenuated strains both in vitro and in vivo [14,15]. 

Still, historically, a major criticism against the use of LAVs is the possibility of recombination with circulating WT viruses [23]. Furthermore, DIVA markers would be rendered useless if they were lost or passed to a WT strain via recombination. In endemic areas, there is evidence of FMDV transmission between wild animals and domesticated livestock and, since the late 2000s, next-generation sequencing (NGS) and phylogenic analysis have demonstrated extensive recombination within circulating FMDV strains [24,25,26]. The mechanism of *Picornavirus* recombination is believed to take place by way of template switching during genome replication; this involves the movement of the RdRp from one parental RNA genome (donor) to another genome (acceptor) midway through negative strand synthesis, thereby yielding a hybrid product [27,28]. Recombination hotspots have been identified throughout the genome with a substantial number in the conserved regions encoding NS genes [26,29,30,31,32,33]. More recently, both intra- and inter-serotypic recombination was observed after experimental infection of natural hosts [34,35,36]. Furthermore, it was demonstrated that recombination occurred during the carrier phase of superinfected cattle but not during acute co-infection [36].

The recombination of FMDV has been studied in cell culture since the 1960s using temperature-sensitive and replication-inhibitor resistant mutants [37,38,39,40,41,42]. More recently, a molecular technique was developed to quantify the levels of recombination in vitro for two positive-sense RNA viruses within the *Picornaviridae* family, poliovirus and senecavirus A [43,44,45]. This assay involves the co-transfection of cells with equimolar amounts of two non-infectious in vitro synthesized RNA templates. One template includes a deletion of three amino acids in RdRp’s active site (ΔGDD), rendering it non-replicative. The other contains deletions in the capsid-encoding region (ΔP1), without which the virus cannot be encapsulated and transmitted. Thus, viable virus can only be generated through genomic recombination events, i.e., the RdRp undergoes template switching from the replication competent donor template (ΔP1) to the replication incompetent acceptor template (ΔGDD), successfully complementing the deleted regions without the generation of additional lethal mutations during negative-strand replication.

In this study, the above methodology was developed for use with A24-FMDV to quantitate the levels of recombination in cell culture using two in vitro synthesized constructs: the donor template A24-ΔP1 (ΔP1) and the acceptor template A24-ΔGDD (ΔGDD). After verifying the validity and reproducibility of the assay, the effects of deoptimization on recombination within the conserved P2P3 region were tested by replacing the donor template ΔP1 with A24-ΔP1-P2P3Deopt (ΔP1-P2P3Deopt). A24-ΔP1-P2P3Deopt is based on the previously described LAV DIVA candidate strain A24-P2P3Deopt [14]. Following transfection of the two genetically engineered non-infectious RNAs, viable recombinant viruses from various time points were plaque purified and analyzed by NGS to identify the general sites of recombination and the stability of the P2P3 deoptimized regions post-recombination. The results highlighted that our in vitro assay was functional. Additionally, codon deoptimization of most of the P2P3 coding region did not completely prevent recombination, although recombinants that contained large regions of deoptimized sequences, including DIVA markers, were less fit than WT in vitro. 

## 2. Materials and Methods

Cells. BHK-21 (Baby hamster kidney [C-13] CCL-10, ATCC) cells were grown in minimum essential media (MEM; Gibco Waltham, MA, USA) supplemented with 10% Fetal Bovine Serum Characterized (HyClone, Logan, UT, USA), 1% Anti-Anti (Gibco, Waltham, MA, USA), 1% MEM Non-Essential Amino Acids (Gibco, Waltham, MA, USA), and 10% Tryptose Phosphate Broth (Teknova, Hollister, CA, USA) at 37 °C in a 5% CO_2_ atmosphere. LFBK-αvβ6 (Fetal porcine kidney expressing the bovine αVβ6 integrin (canonical FMDV receptor)) cells were grown in Dulbecco’s modified Eagle’s medium (DMEM; Gibco, Waltham, MA) supplemented with 10% Fetal Bovine Serum Characterized, 1% Anti-Anti, and 1% MEM Non-Essential Amino Acids at 37 °C in 5% CO_2_ atmosphere [46]. 

Plasmid Construction and RNA Synthesis. pA_24_Cru and pA_24_Cru-*Nhe*I P2P3Deopt have been previously described [14]. pA24-ΔP1 was constructed by replacing the P1 coding region of pA_24_Cru-(containing unique *Nhe*I and *Fse*I) with a 60 bp de novo synthesized fragment (GenScript, Piscataway, NJ, United States) that contained 15 bp of VP4 directly followed by 27 bp of VP1 via subcloning (Figure S1) [47]. pA24-ΔP1-P2P3Deopt was subsequently constructed from this vector by replacing the P2P3 fragment with the equivalent fragment from the previously described pA_24_Cru-*Nhe*I P2P3Deopt [14]. pA24-ΔGDD was constructed by replacing a *BamH*I*/Swa*I fragment of pA_24_Cru with a 925 nt de novo synthesized fragment (GenScript, Piscataway, NJ, United States) that contained the 9-nucleotide deletion of the GDD active site of 3D (Figure S1). Modified plasmids were digested with *Swa*I for linearization and viral RNAs were synthesized by in vitro transcription with T7 polymerase using a MEGAscript T7 kit (Thermo Fisher, Waltham, MA, USA) digested with the included TurboDNase and purified with a RNeasy kit (Qiagen, Hilden, Germany) following the manufacturer’s directions. RNA quantification was performed on a Nanodrop Spectrophotometer ND 1000, and integrity was analyzed using an Agilent Bioanalyzer (Santa Clara, CA, USA).

Cell-Based Recombination Assay. BHK-21 cells were seeded at 1 × 10^6^ cells/well in a 6-well tissue culture dish (Costar or Corning, Corning, NY, USA). Following an overnight incubation, cell media was replaced by 500 µL of complete cell culture media. Cellular monolayers were then transfected with 1.7 × 10^11^ copy number of the indicated RNA (equivalent to 580–781 ng) in 15 µL of Lipofectamine 2000 reagent (Invitrogen, Waltham, MA, USA) diluted in 250 μL of Opti-MEM (Gibco, Waltham, MA) for 2–3 biological repeats per reaction. Complete media (1.5 mL) was added to the wells after a 3 h incubation at 37 °C in 5% CO_2_. Plates were incubated at 37 °C in 5% CO_2_ until frozen at 24 or 48 hpt.

Plaque Assay and Plaque Isolation. Virus from thawed cell lysates was titered in duplicate in LFBK-αvβ6 cells. Plaques were visualized by staining cell cultures with crystal violet, which was followed by quantitation at 48 hpt. Plaque isolation was performed for each biological repeat of the ΔP1-P2P3Deopt × ΔGDD recombinant product as previously described [35]. Briefly, 200 µL of diluted cell lysate was incubated for 1 h at 37 °C in 5% CO_2_, which was followed by the addition of 2 mL of agarose overlay media (1:1 ratio of 2% UltraPure Low Melting Point Agarose (Invitrogen; Thermo Fisher, Waltham, MA, USA) and 2× MEM (Gibco, Waltham, MA, USA)), supplemented with 1% Anti-Anti and 1% MEM Non-Essential Amino Acids. At 48 hpt, 200 µL of clearly detected isolated plaques were randomly chosen, quickly collected before agar solidification, diluted in 100 µL of PBS++ (Gibco, Waltham, MA, USA) and frozen. Specific plaques were chosen, which was followed by passing in monolayers of LFBK-αvβ6 in T25 flasks, and they were frozen after detection of CPE (24–48 hpt). 

Viral RNA Extraction, Sequencing, and NGS analysis. FMDV full-length genome sequencing was performed as previously described [36]. In brief, FMDV RNA was extracted from either isolated plaques or total cell culture supernatants using the MagMAX Viral RNA Isolation Kit (Thermo Fisher, Waltham, MA, USA), reverse-transcribed using Superscript II (Invitrogen; Thermo Fisher, Waltham, MA, USA) using random hexameric primers, followed by Nextera XT (Illumina, San Diego, CA, USA) library construction. Reads were filtered for quality and length and competitively mapped to either A24 Cruzeiro (GenBank #AY593768) or A24-P2P3Deopt DIVA in CLC Genomics Workbench v. 21 (Qiagen; Hilden, Germany) [14].

Statistical Analysis. Data handling, analysis, and graphic representations were performed using Prism 9 (GraphPad Software, San Diego, CA, United States), Microsoft Excel (Microsoft, Redmond, WA, United States) and RStudio 4.1.2 (RStudio Team (2020). RStudio: Integrated Development for R. RStudio, PBC, Boston, MA, USA). Statistical significance was determined using one-tailed Student’s *t* test; *p* values of <0.05 were considered significant.

## 3. Results

**Figure 1:**
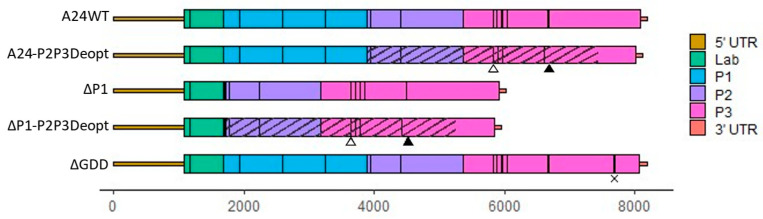
Schematic diagram of the FMDV RNA templates used during transfection. DIVA markers are indicated by open (3B1 deletion, RQKP9-12→PVKV) and closed (3D H27N31→YR) triangles. Deoptimized regions are indicated by stripes. ΔGDD within the active site of RdRp encoded by 3D is indicated by an X.

**Figure 2:**
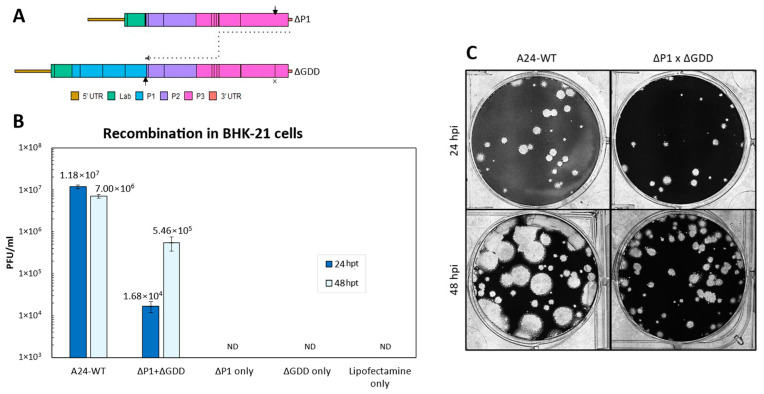
(**A**). Schematic model of FMDV recombination during negative-strand synthesis with the given templates. ΔGDD within the active site of RdRp encoded by 3D is indicated by an X. Template switching must occur between the two arrows. (**B**). Number of infectious particles generated 24 and 48 h post-transfection in BHK-21 cells. Error bars represent 1 standard deviation. ND—not detected. (**C**). Plaque morphologies of WT and recombinants in LFBK-αVβ6 cells stained at 48 hpt.

Recombination Rates of ΔP1 and ΔGDD. To quantify recombination, a cell culture-based assay previously developed for two members of the *Picornaviridae* family, poliovirus and senecavirus A, was implemented for FMDV [43,44,45]. The FMDV serotype A24Cru infectious clone (pA24-Cruz) [48] was modified to create two vectors (Figure 1 and Table 1): (a) pA24-ΔP1 has a 2172 in-frame nucleotide deletion of the P1 coding region that leaves the first five amino acids of VP4 and the last nine amino acids of VP1 intact; (b) pA24-ΔGDD contains a nine-nucleotide deletion that removes the GDD motif from the active site of 3D, rendering RdRp unable to replicate the viral genome. Viral RNA was synthesized from both vectors to create the genomic templates ΔP1 and ΔGDD and from an unmodified pA24-Cru to create the A24-WT genome. To produce viable virus, recombination must follow the diagram detailed in Figure 2A. In short, the 3D RdRp coding region must be translated from the ΔP1 template. During negative-strand synthesis, RdRp must switch templates from the ΔP1 donor to the ΔGDD recipient between the two indicated black arrows (~3800 nt window, Figure 2A) to successfully synthesize a negative-strand template without any deletions or additional lethal mutations (Figure 2A). As expected, the transfection of A24-WT RNA produced viable infectious particles at 24 hpt (1.2 × 10^7^ plaque-forming units (PFU)/mL ± 1.3 × 10^6^) and 48 hpt (7.0 × 10^6^ PFU/mL ± 7.5 × 10^5^) and, as expected, single transfections of either non-infectious ΔP1 and ΔGDD RNAs failed to produce infectious particles (Figure 2B). In contrast, infectious particles were successfully generated following the co-transfection of both non-infectious RNAs (donor) ΔP1 and (recipient) ΔGDD. The appearance of infectious viral particles indicated successful recombination, although lower viral titers were derived by using these modified RNA molecules compared to the unmodified WT control RNAs at 24 hpt (1.7 × 10^4^ ± 5.1 × 10^3^ PFU/mL) and 48 hpt (5.5 × 10^5^ ± 2.1 × 10^5^ PFU/mL). Viral plaque morphologies were similar for WT and ΔP1 × ΔGDD recombinants examined at 24 hpt; however, by 48 h, the WT plaques were larger than those observed in the recombinants, confirming adequate fitness for viruses derived from the WT template (Figure 2C) [49]. The above assays indicate that recombination between non-infectious FMDV RNAs generated infectious virus that allowed for quantification in PFU/mL in vitro.

**Figure 3:**
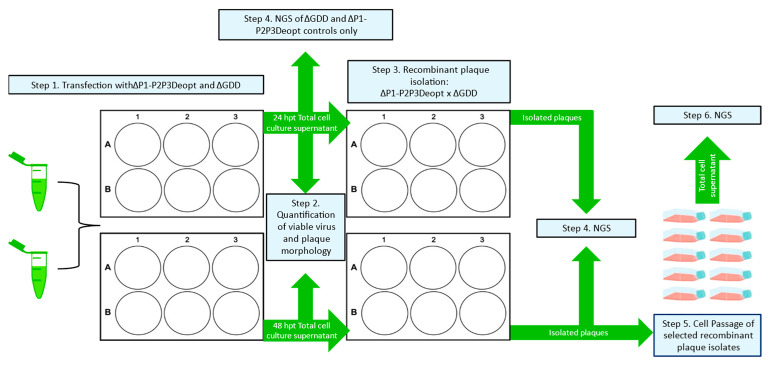
Experimental design schematic of the ΔP1-P2P3Deopt × ΔGDD recombination assay and the proceeding viral quantification, plaque isolation, plaque passing, and sample types that were sequenced by NGS.

**Figure 4:**
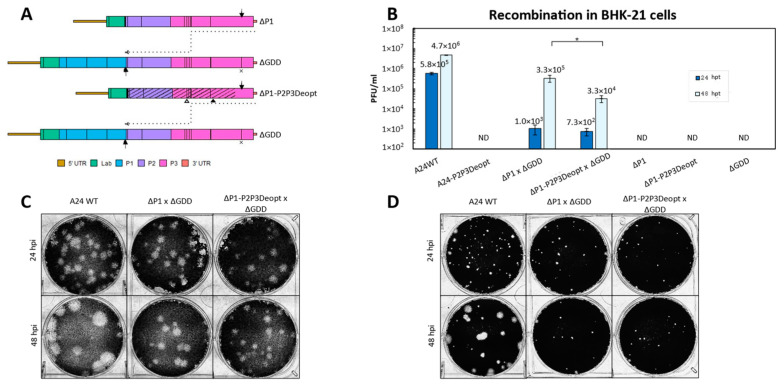
(**A**). Schematic model of FMDV recombination during negative-strand synthesis with the given templates. DIVA markers are indicated by open (3B1 deletion, RQKP9-12→PVKV) and closed (3D H27N31→YR) DIVA markers. Deoptimized regions are indicated by stripes. The ΔGDD within the active site of RdRp encoded by 3D is indicated by an X. Template switching must occur between the two arrows. (**B**). Number of infectious particles generated 24 and 48 h post-transfection in BHK-21 cells. * Statistically significant difference between the number of infectious virions produced from the different donor RNA templates. Error bars represent 1 standard deviation. ND—not detected. (**C**,**D**). Plaque morphologies of WT and recombinants in BHK-21 or LFBK-αVβ6 cells respectively, stained at 48 hpt.

Recombination efficiency of ΔP1-P2P3Deopt and ΔGDD. Previously, it was demonstrated that codon deoptimization of the conserved non-structural coding P2 and P3 regions of FMDV A24Cru gave rise to attenuated viruses in cell culture, laboratory animal models, and natural hosts including swine [14] and cattle (unpublished). To determine if CD of the P2-P3 genomic regions would prevent the likelihood of recombination with respect to its parental non-deoptimized clone, the pA24-ΔP1 template mentioned above was modified to contain P2-P3 deoptimized coding sequences. Of note, although highly codon deoptimized, this construct was designed to contain stretches of non-deoptimized (WT) sequences, which were purposely preserved to maintain the predicted RNA secondary structure contained within those regions (Figure S2). RNA transfections followed the diagram detailed in Step 1 of Figure 3; the generation of a negative-strand template via successful template switching that is suitable to generate infectious particles occurs as detailed in Figure 4A. The transfection of A24-WT RNA produced viable infectious particles at 24 hpt (5.8 × 10^6^ ± 8.5 × 10^4^ PFU/mL) and at 48 hpt (4.7 × 10^6^ ± 1.3 × 10^5^ PFU/mL). No infectious particles were generated when the non-infectious RNA templates (ΔP1, ΔP1-P2P3Deopt, or ΔGDD) were transfected alone (Figure 4B). Unexpectedly, the transfection of full-length control A24-P2P3Deopt RNA did not produce infectious virus at 24 or 48 hpt; granted, the original A24-P2P3Deopt virus previously characterized in cell culture and in animals was derived after the transfection of 10,000 ng of RNA, utilized different transfection conditions (electroporation), and required several rounds of blind passes in BHK-21 cells before viable virus was observed [14]. No significant differences (*p* = 0.26) were detected between the number of ΔP1 × ΔGDD (1.3 × 10^3^ ± 5.4 × 10^2^ PFU/mL) and ΔP1-P2P3Deopt × ΔGDD (7.2 × 10^2^ ± 2.9×10^2^ PFU/mL) infectious recombinants generated at 24 hpt (Figure 4B). Additionally, plaque morphologies were similar between WT and the ΔP1 × ΔGDD recombinants and ΔP1-P2P3Deopt × ΔGDD recombinants at 24 hpt in BHK-21 (Figure 4C) and in porcine LFBK-αvβ6 cells (Figure 4D). Increased viral production at a later time point was detected in recombinants derived from non-deoptimized donor sequences. Specifically, at 48 hpt, there was a statistically significant 10-fold difference between the number of infectious particles generated by ΔP1 × ΔGDD (3.3 × 10^5^ ± 1.2 × 10^5^ PFU/mL) and ΔP1-P2P3Deopt × ΔGDD (3.3 × 10^4^ ± 1.3 × 10^4^ PFU/mL) (*p* = 0.03). At 48 hpt, plaque morphologies were again similar between the ΔP1 × ΔGDD recombinants and ΔP1-P2P3Deopt × ΔGDD recombinants in both BHK-21 and LFBK-αvβ6 cells; however, WT had larger plaques than either recombinant. These results indicated that in our system, the presence of deoptimized sequences in donor RNA P2 and P3 regions fails to abolish recombination when examined at 24 or 48 hpt.

**Figure 5:**
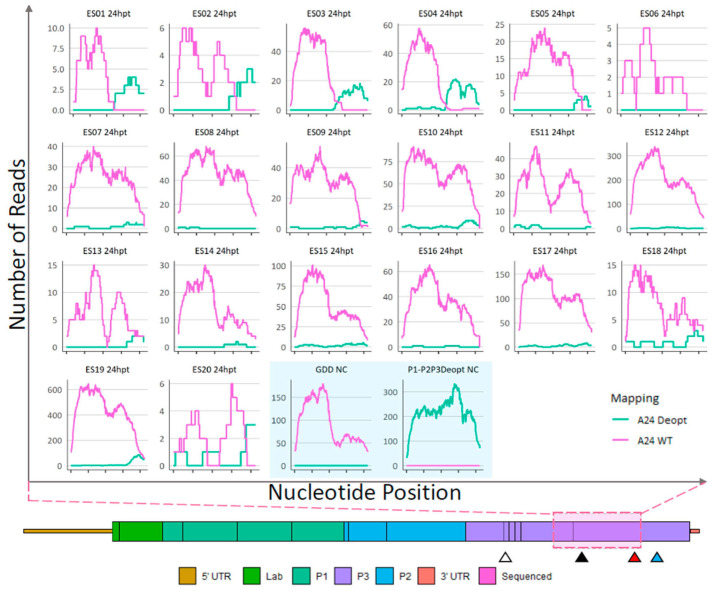
Sequence analysis of 24-hpt ΔP1-P2P3Deopt × ΔGDD recombinant plaque isolates and ΔP1-P2P3Deopt or ΔGDD transfection total cell supernatant (shaded blue box). Locations (*x*-axis) and counts (*y*-axis) of reads that preferentially map to either a A24-WT or A24-P2P3Deopt reference within a 1059 nt section of the 3CD coding region as indicated by the pink shaded box. Triangles indicate the location of the 3B DIVA marker (white), 3D DIVA marker (black), the boundary between deoptimized and non-deoptimized regions (red), and the GDD deletion (blue).

**Figure 6:**
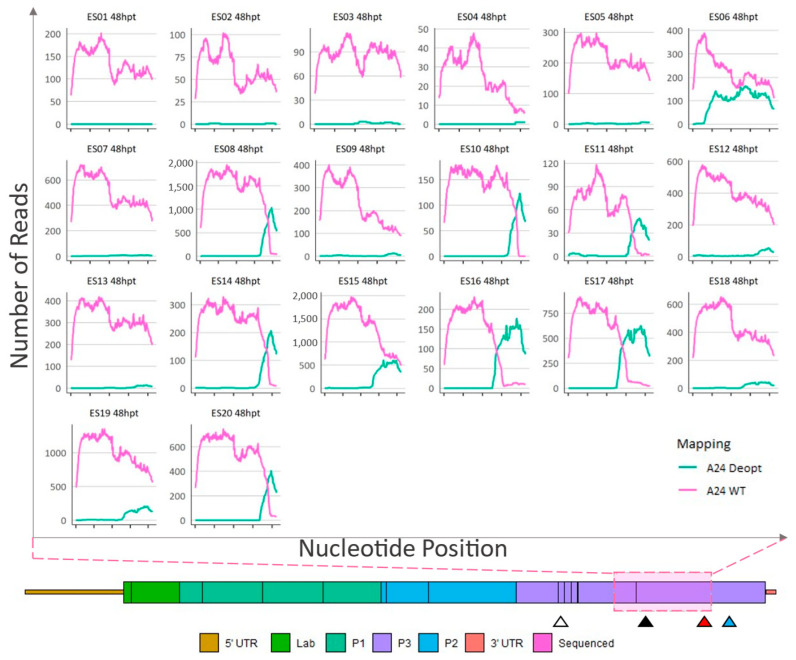
Sequence analysis of 48-hpt ΔP1-P2P3Deopt × ΔGDD recombinant plaque isolates. Locations (*x*-axis) and counts (*y*-axis) of reads that preferentially map to either a A24-WT or A24-P2P3Deopt reference within a 1059 nt section of the 3CD coding region as indicated by the pink shaded box. Triangles indicate the location of the 3B DIVA marker (white), 3D DIVA marker (black), the boundary between deoptimized and non-deoptimized regions (red), and the GDD deletion (blue).

**Figure 7:**
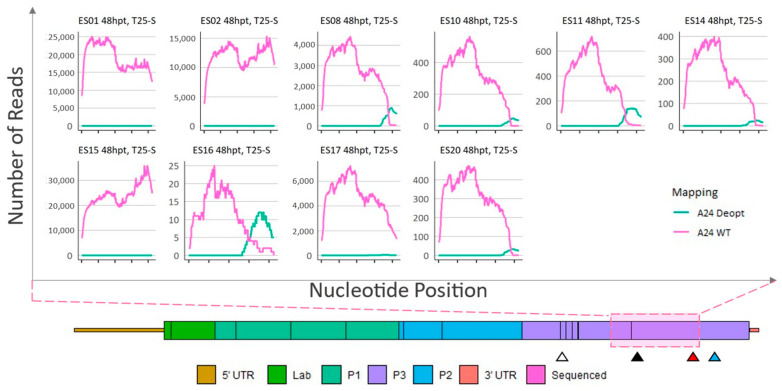
Sequence analysis of total cell culture supernatant of 48-hpt ΔP1-P2P3Deopt × ΔGDD recombinants plaque isolates passed in LFBK-αvβ6 cells. Locations (*x*-axis) and counts (*y*-axis) of reads that preferentially map to either a A24-WT or A24-P2P3Deopt reference within a 1059 nt section of the 3CD coding region as indicated by the pink shaded box. Triangles indicate the location of the 3B DIVA marker (white), 3D DIVA marker (black), the boundary between deoptimized and non-deoptimized regions (red), and the GDD deletion (blue).

NGS analysis of recombinants. As detailed in Figure 3 (Steps 4 and 6), RNA extractions followed by Illumina sequencing was performed to assess the sites of recombination in ΔP1-P2P3Deopt × ΔGDD recombinant plaque isolates originating from the 24 hpt (Figure 5 and Figure S3), and the 48 hpt cell lysates (Figure 6), and also from selected 48 hpt plaques passed in LFBK-αvβ6 cells (Figure 7). Non-infectious ΔP1-P2P3Deopt and ΔGDD transcribed templates were included as controls. Following full-length genome sequencing, using CLC Genomics Workbench, reads were competitively mapped to three reference genomic regions that represented either the WT or deoptimized reference genomes: a 256 nt region of 2A and 2B, a 1413 nt region including 2C and 3A, and a 1059 nt region of 3C and 3D (graphically illustrated in Figure S3). The depth of coverage for reads that preferentially mapped to the WT (pink) or deoptimized (green) reference at each position are indicated in the graphs (Figure 5, Figure 6, Figure 7 and Figure S3). As expected, reads derived from supernatant transfected with the RNA templates ΔP1-P2P3Deopt or ΔGDD mapped exclusively to the transfected template (Figure 5). NGS revealed that all plaques isolated from the 24 hpt sample had recombinant genomes with at least sub-consensus levels of deoptimized sequence; however, most of the genomes within twelve plaques (ES06…08, ES10…12, and ES-14…ES-19) were composed of a WT sequence at the consensus level at all locations within the genome (Figure 5 and Figure S3). Only three plaques (ES10, ES17, and ES19) isolated from the 24 hpt transfection had an extremely low amount (maximum depth of coverage of 2 reads/position) of sub-consensus deoptimized sequence present in the 2AB region (Figure S3). Comparatively, the average read depth mapped to the corresponding WT sequence within each sample ranged between 38 and 253 reads/nt. Similarly, within the 2C-3A region, fifteen plaques isolated from the 24 hpt transfections had low levels of sub-consensus deoptimized sequence (Figure S3). Within the 3CD coding region, evidence of recombinants composed of genomes with mixed WT and deoptimized sequence was much more apparent with all but one plaque (ES06) containing deoptimized sequence. Indeed, multiple plaque isolates from the 24 hpt transfection (ES01…05, ES09, and ES20) contained a deoptimized sequence at the consensus level at the 3′ end of the 3CD region (Figure 5).

Plaques isolated from the 48 hpt transfection did not contain any deoptimized sequence in the 2AB or 2C-3A region (data not shown). Like the plaques isolated from samples acquired at 24 hpt, multiple sites of obvious recombination were recorded in the 3CD region and, within the 3′ end of the indicated area, plaques ES08, ES10, ES11, ES14, ES16, ES17, and ES20 were composed of a deoptimized sequence at the consensus level (Figure 6). In contrast, as previously seen with plaques isolated at 24 hpt, the genomes within plaques ES01, ES02, ES03, ES04, ES05, ES06, ES07, ES09, ES12, ES13, ES15, ES18, and ES19 were composed of consensus-level WT sequences in the entire 1059 nt region (Figure 6). 

To determine the stability of the deoptimized sequences, plaques ES08, ES10, ES11, ES14, ES15, ES16, ES17, and ES20 isolated from the 48 hpt infection were passed in cell culture. Plaques ES01 and ES02 were also included as a control. Total cell culture supernatants were collected, and viral RNA was sequenced and analyzed as detailed above. As expected, after passage, samples ES01 and ES02 remained almost exclusively WT (Figure 7). Interestingly, plaques ES15 and ES17 were composed of consensus-level WT sequence losing all but an extremely low level of deoptimized sequences (relative to WT 0.01% for ES15 and 0.16% for ES17). In contrast, the other samples (ES08, ES10, ES11, ES14, ES16, and ES20) retained the deoptimized codons. Furthermore, the breakpoint nt position in these samples (ES08, ES10, ES11, ES14, ES16, and ES20) remained within 3–80 nt of the previously observed boundaries (Table S1), and the relative depth of coverage between WT and deoptimized reads did not change, indicating that these samples did not gain WT or deoptimized sequences. 

## 4. Discussion

The work presented herein demonstrates that the in vitro recombination assay previously developed for poliovirus and senecavirus A can be adapted to quantify and evaluate recombination events for FMDV [43,44,45]. 

The co-transfection of ΔP1-P2P3Deopt × ΔGDD produced approximately the same number of viable particles as co-transfection of ΔP1 × ΔGDD RNAs at 24 hpt, indicating that recombination could still occur despite the presence of CD sequences. Furthermore, our data suggest that even if recombination occurred between a WT strain and the A24-P2P3Deopt LAV candidate in vitro, the resulting recombinant would not display a gain of fitness. Illumina sequencing of the ΔP1-P2P3Deopt × ΔGDD recombinants revealed that at 24 or 48 hpt, most recombinants were fully composed of WT sequences at the consensus level and only very few recombinants contained deoptimized sequences at the consensus level. Furthermore, even at the sub-consensus level, recombinants were much more likely to contain deoptimized sequences in the 3CD coding region, as only a few recombinants isolated at 24 hpt contained deoptimized sequences in the 2AB and 2C-3A coding regions (Figure S3). Thus, our assay demonstrates that the DIVA markers were not passed and maintained; all recombinants that contained consensus level CD contained the deoptimized sequence downstream of the DIVA markers. Of note, only one recombinant (ES06) isolated at 48 hpt contained the 3D DIVA markers at an appreciable coverage, although it was still at a sub-consensus level. There are conflicting reports regarding the influence of sequence homology in viral recombination [50]. Thus, the absence of plaque isolated recombinants containing consensus levels of deoptimized sequences in 2AB and 2C-3A coding regions may not be simply linked to a decrease in sequence homology compared to WT. More likely, recombinants did not contain deoptimized sequence at the consensus level in the 2AB and 2C-3A coding regions because large regions of deoptimization can cause an attenuation in FMDV [14,15]. The assay was specifically performed in a cell line that lacked an intact innate immune system to limit the number of host-associated variables. Still, A24-P2P3Deopt virus has been shown to have a slower growth rate in BHK-21 cells compared to the parental WT [14]. How codon deoptimization affects a virus is a very complex issue and has not been thoroughly examined in FMDV. In other viruses, codon deoptimization has been shown to cause a decrease in the rate of translation, aberrant folding of the nascent amino acid chain during translation, a decreased replication rate, an increase in dinucleotide frequencies CpG and UpA, and destabilization of conserved RNA structures [17]. Perhaps a combination of these factors affected the recovery of viable A24-P2P3 deoptimized, but not A24 WT viruses, after the transfection of full-length viral RNA in our in vitro assay. Furthermore, the ability to generate a less deoptimized genome via recombination of the two non-infectious RNA molecules (ΔP1-P2P3Deopt × ΔGDD RNA) may have facilitated template switching and the overall rate of replication and translation required for the generation of viable virus from non-defective full genomes [51]. Hence, the absence of plaque isolated recombinants containing large regions of deoptimized sequence at the consensus level could have been caused by a number of different and difficult to dissect molecular mechanisms, the elucidation of which, was beyond the scope of this study. Future studies targeted to understand those mechanisms will hopefully answer these questions.

Due to the molecular biology of FMDV, certain limitations were inherent to the study. In the recoding design of the original A24-P2P3deopt virus, codon deoptimization of FMDV P2 and P3 was excluded from regions that were predicted to form highly conserved secondary RNA structures. These regions have been confirmed to be critical for viral genome replication, irrespective of FMDV serotype [52]. Within the 3D coding region, only the first 272 of the 470 total codons were deoptimized. Accordingly, ΔP1-P2P3Deopt and ΔGDD contain a 185 nt homologous sequence between the boundary of deoptimized 3D and the ΔGDD site. If recombination were to occur in this region, a genome containing only WT sequence would be generated. We could further conclude that the emergence of WT sequence via the re-optimization of all deoptimized codons is extremely unlikely. In fact, we have previously demonstrated that the genome of the A24-P2P3Deopt virus was stable after several passages in vitro [14].

Alternatively, instead of the formation of recombinants with full-length genomes, the generation of infectious particles could be explained by the presence of defective interfering (DI) particles, which is a phenomena that has been previously described for FMDV [53]. Noteworthy, these DI particles only became apparent after multiple passages (over 200) in cell culture without innate immune pressure. In our study, the assay determines the products of recombination after only one blind passage post-RNA transfection in cell culture. Furthermore, the absence of reads containing either ΔP1 or ΔGDD sequences suggests that no DI particles were present in our samples. 

In our assay, we cannot assume that all infectious particles titered at 24 or 48 hpt arose from a unique transfection event. Given the fast replication kinetics of FMDV, it is possible that infectious particles generated early post-transfection underwent multiple rounds of replication, thus inflating the total observed number of viable viruses. While plaque sequencing results indicate that all ΔP1-P2P3Deopt × ΔGDD RNA recombinants contained unique genome sequences, i.e., different breakpoints, a much larger sampling size would be needed to definitively support this hypothesis. An additional complication is that multiple viruses can contribute to a single viral plaque formation. This could result in the observation of multiple sub-consensus genomes within the purified plaques [54]. Lastly, recombination has been described as a frequent and continuous process during viral replication for picornaviruses and other positive-sense RNA viruses [50,55]. Although FMDV has not been investigated as deeply as other picornavirus family members, a similar situation could also explain the detection of multiple sub-consensus genomes in the isolated FMDV recombinants. 

Additional time points and NGS that produces long reads could further refine our assay. As demonstrated in poliovirus, when imprecise recombination occurs, duplications within the genome are generated. Additional recombination events can later refine and correct the introduced errors, thus generating fitter viruses [56]. For FMDV, the NGS data indicate that even when a large portion of the viral genome sequence was deoptimized, recombination could still occur within relatively short unchanged sequence stretches, deriving fitter WT genomes. It is unclear from our limited sampling if the full-length WT virus emerged from one or multiple recombination events within the homologous region to eventually outcompete the recombinants containing deoptimized sequence or if, alternatively, the recombinants containing deoptimized sequence gained more WT sequence through multiple recombination events.

Additionally, in its current iteration, our assay could only quantify and sequence recombination events that should have resulted in viable virus. However, based on the reported frequency of recombination during replication of poliovirus [56], it is likely that a higher number of recombination events may occur within FMDV sequences, as compared to those detected in this study. 

It is possible that the recently described RNA secondary structures within 3D contributed to the higher levels of recombination in the non-deoptimized 3D region [52]. Indeed, in the 1980s, early software predicted the formation of RNA stem loop structures in recombination hotspots observed during FMDV recombination in cell culture; however, other more recent studies have demonstrated conflicting data in other viruses [50,57]. Thus, it is unclear if the essential RNA structures in 3D contributed to a global pattern of higher recombination frequency in that area or if the potential decrease in the stability of RNA structures within the P2P3 region may have contributed to a lower level of recombination in those areas. Additional studies are needed to determine the role of RNA secondary structures during FMDV recombination. 

Although informative, care must be taken when interpreting the results from the assay presented in this manuscript and linking it to what could occur during a co-infection of two strains of FMDV in a host. Deoptimization of the P2 and P3-coding regions resulted in attenuated phenotypes in at least two FMDV susceptible hosts of importance, swine, and cattle [14] and unpublished]. Of course, the in vitro assay described in this manuscript does not consider any recombination that would allow for the newly generated virus to escape the innate or adaptive immune system of the host (e.g., swapping capsids, gaining mutations that improve fitness, etc.) nor has attenuation through the codon deoptimization of the P2-P3 coding region in different serotypes been demonstrated. Notwithstanding, a backbone of A24 P2P3 with DIVA markers has been shown to be a reliable platform and vaccine candidate, allowing for the insertion of different serotype specific capsids [47].

## 5. Conclusions

Taken together, our data indicate that: (1) an in vitro assay to measure recombination for FMDV genome sequences was successfully established; (2) the majority of observed recombinants were generated by template switching that presumably occurred positionally very early during the reading of the template strand, either within the 185 nt of non-deoptimized sequence or within the first 750 nt of the 3507 nt deoptimized region in the negative strand; (3) in our system, codon deoptimization did not completely prevent recombination; (4) however, recombinants containing large regions of deoptimized regions at the consensus level were not observed and presumably displayed lower fitness than WT. Thus, our results suggest that deoptimization of the P2 and P3 coding regions of FMDV is an effective strategy to cause viral attenuation and—at least in our in vitro system—prevent the passing of DIVA markers between intra-serotype genomes. This information may be considered in the development of subsequent generations of LAVs for FMDV.

## Figures and Tables

**Table 1:** Genotypic and phenotypic characteristics of viral RNAs.

Template	Infectious	Replication Competent
A24WT	✓	✓
A24-P2P3Deopt	✓	✓
ΔP1	×	✓
ΔP1-P2P3Deopt	×	✓
ΔGDD	×	×

## Data Availability

The original contributions presented in the study are included in the article/Supplementary Material, further inquiries can be directed to the corresponding author.

## Supplementary Materials

The following supporting information can be downloaded at: https://www.mdpi.com/article/10.3390/v15030670/s1, Figure S1: ΔP1 and ΔGDD Cloning Fragments; Figure S2: Alignment of the P2P3 coding region originating from A24WT and A24-P2P3Deopt; Figure S3: Sequence Analysis of 24-hpt ΔP1-P2P3Deopt x ΔGDD Recombinant Plaque Isolates; Table S1: Recombinant Breakpoint Location (nt) within the 3CD Coding Region.
