# Supplementary material for: Evaluation of Potential In Vitro Recombination Events in Codon Deoptimized FMDV Strains
Source: Viruses. 2023 Mar 2;15(3):670. doi: 10.3390/v15030670 (PMC10052203; doi:10.3390/v15030670)
Supplement: Supplementary file 1 [file viruses-15-00670-s001.zip › Supplementary-Table_01.docx]

| Recombinant Breakpoint^a^ Location (nt) within the 3CD Coding Region | | |
| --- | --- | --- |
| Sample | Plaque isolate (48 hpt) | Total cell culture supernatant |
| ES08 | 939 | 943 |
| ES10 | 939 | 945 |
| ES11 | 830 | 854 |
| ES14 | 932 | 851 |
| ES16 | 687 | 765 |
| ES17 | 704 | NA |
| ES20 | 941 | 956 |
| ^a^breakpoint is defined as the first nucleotide position where the coverage of deoptimized reads is greater than the coverage of WT reads | | |
