# Supplementary material for: Evaluation of Potential In Vitro Recombination Events in Codon Deoptimized FMDV Strains
Source: Viruses. 2023 Mar 2;15(3):670. doi: 10.3390/v15030670 (PMC10052203; doi:10.3390/v15030670)
Supplement: Supplementary file 1 [file viruses-15-00670-s001.zip › Supplementary_01.pdf]

>ΔP1 Cloning fragment

*GGCCGGCC*AATCCATTGCACCAGCAAAGCAGCTTCTGAATTTTGACCTGCTTAA*GCTAGC*

>ΔGDD Cloning Fragment

*GGATCC*TGAAGACTCTCGTGAACACGGAACACGCCTATGAGAACAAACGCATCACTGTTG
AAGGCGGGATGCCATCTGGTTGTTCCGCAACAAGCATCATCAACACAATTTTGAACAACA
TCTACGTGCTCTACGCTTTGCGTAGACACTATGAGGGAGTTGAGCTGGACACTTACACCA
TGATCTCTTACATCGTGGTGGCAAGTGATTACGATTTGGACTTTGAGGCTCTCAAGCCCC
ACTTCAAATCCCTTGGTCAAACCATCACTCCAGCTGACAAAAGCGACAAAGGTTTTGTTC
TTGGTCACTCCATTACTGATGTCACTTTCCTCAAAAGACACTTCCACATGGATTATGGAA
CTGGGTTTTACAAACCTGTGATGGCCTCAAAGACCCTTGAGGCTATCCTCTCCTTTGCAC
GCCGTGGGACCATACAGGAGAAGTTGATCTCCGTGGCAGGACTCGCTGTTCACTCTGGAC
CAGACGAGTACCGGCGTCTCTTCGAGCCCTTTCAAGGCCTCTTCGAGATTCCAAGCTACA
GATCACTTTACCTGCGTTGGGTGAACGCCGTGTGCGGCGACGCATAATCCCTCAGAGACT
ACATTGGCATACTGTTTCTGAGGCGCGCGACGCCGTAGGAGTGAAAAGCCTGAAAGGGCT
TTTCCCGCTTCCTATTCCAAAAAAAAAAAAAAAAAAGCGGCCGGCATGGTCCCAGCCTCC
TCGCTGGCGCCGGCTGGGCAACATTCCGAGGGGACCGTCCCCTCGGTAATGGCGAATGGG
ACGGGGCCGGGCTGCTAACAAAGCCCGAAAGGAAGCTGAGTTGGCTGCTGCCACCGCTGA
GCAATAACTAGCATAACCCCTTGGGGCCTCTAAACGGGTCTTGAGGGGTTTTTTGCTGAA
AGGAGGAACTATATCCGGAGGCCT*ATTTAAAT*
