# Supplementary material for: Evaluation of Potential In Vitro Recombination Events in Codon Deoptimized FMDV Strains
Source: Viruses. 2023 Mar 2;15(3):670. doi: 10.3390/v15030670 (PMC10052203; doi:10.3390/v15030670)
Supplement: Supplementary file 1 [file viruses-15-00670-s001.zip › Supplementary_02.pdf]

Alignment of the P2P3 coding region originating from A24WT and A24 P2P3Deopt

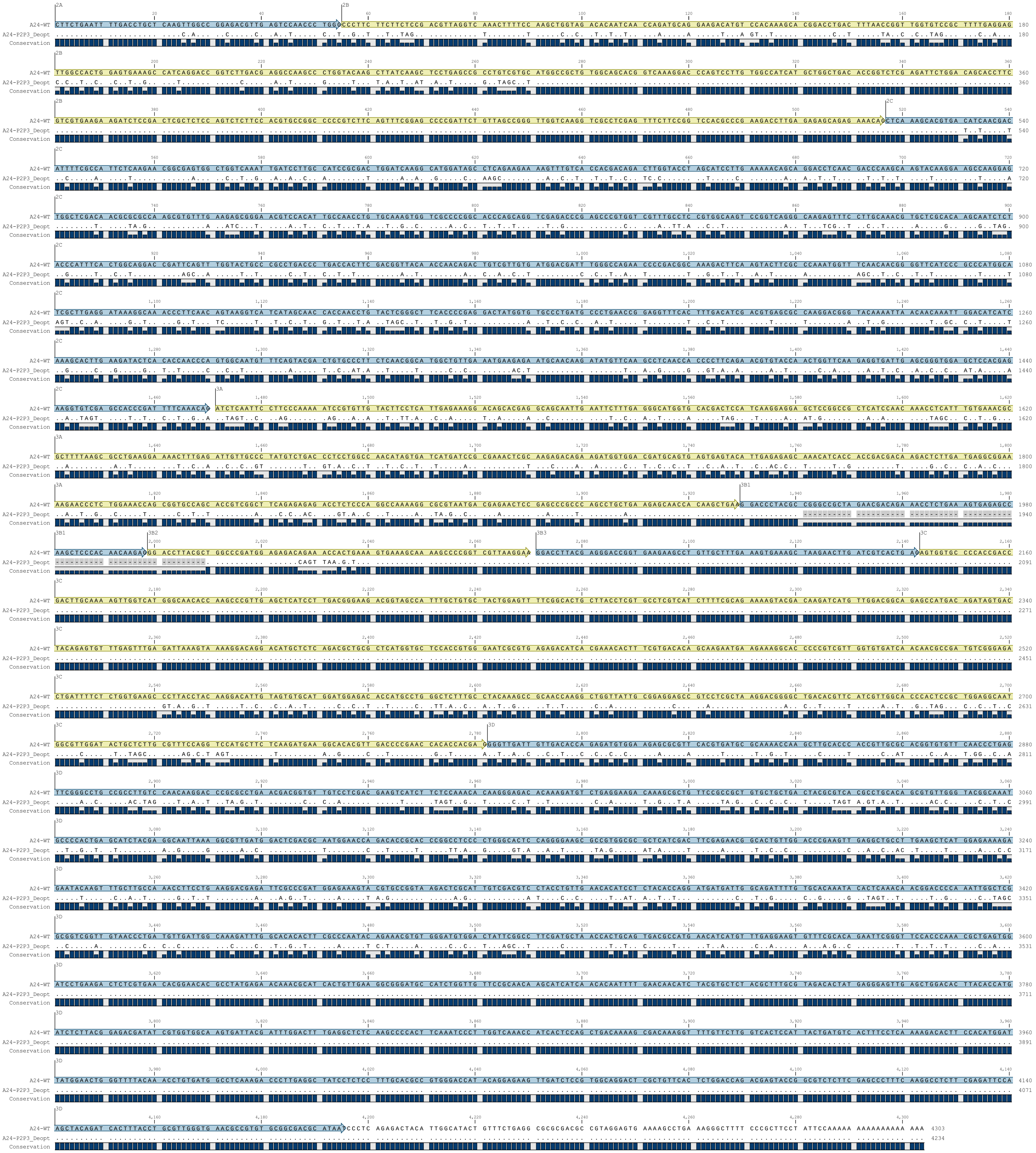

Figure S2. Alignment of the P2P3 coding regions derived from A24WT and A24 P2P3Deopt. ORF of the indicated proteins are represented by alternating blue and yellow arrows.
