# Supplementary material for: Evaluation of Potential In Vitro Recombination Events in Codon Deoptimized FMDV Strains
Source: Viruses. 2023 Mar 2;15(3):670. doi: 10.3390/v15030670 (PMC10052203; doi:10.3390/v15030670)
Supplement: Supplementary file 1 [file viruses-15-00670-s001.zip › Supplementary_03.pdf]

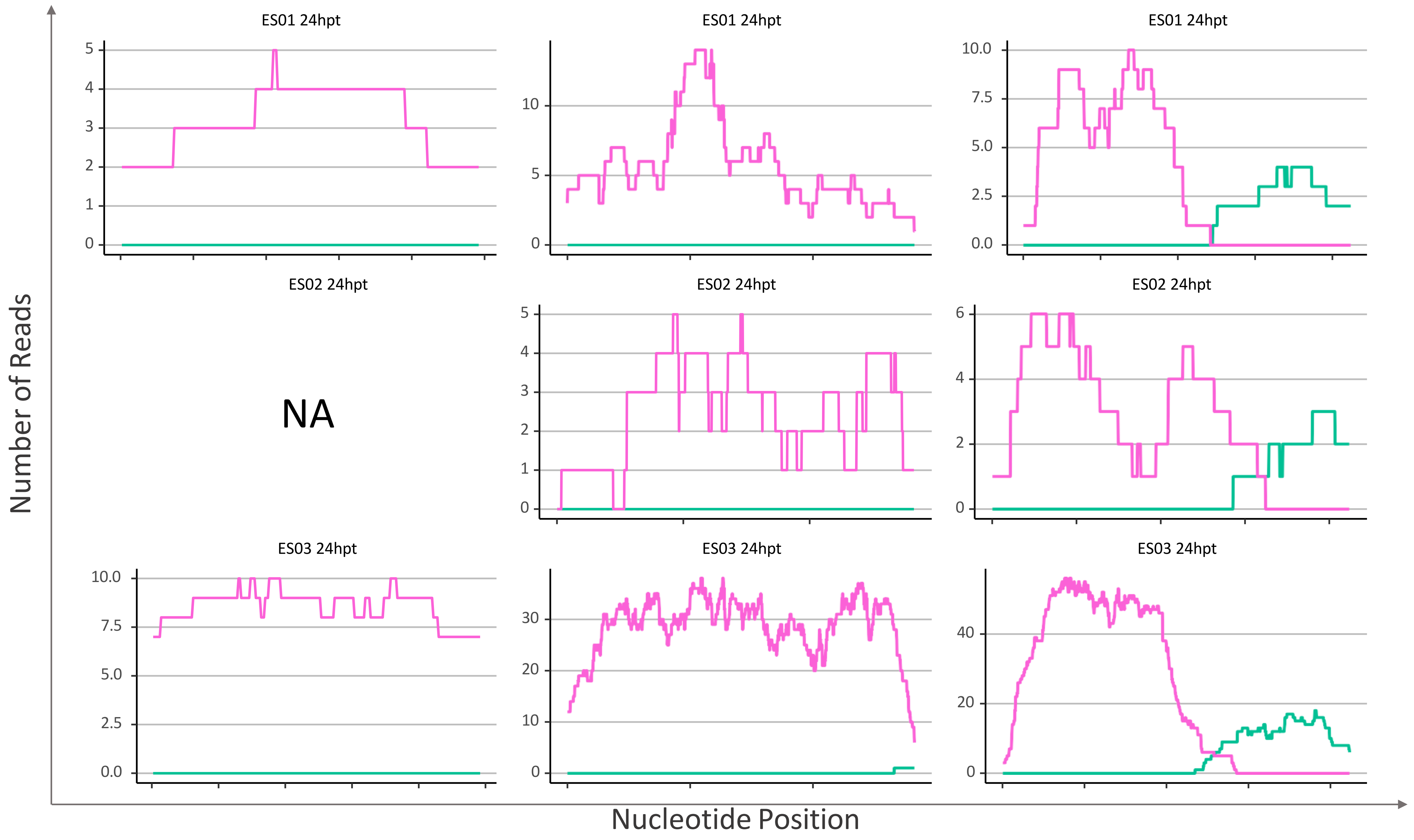

ES01 24hpt
ES01 24hpt
ES01 24hpt
ES02 24hpt
ES02 24hpt
ES02 24hpt
NA
ES03 24hpt
ES03 24hpt
ES03 24hpt
Number of Reads
Nucleotide Position

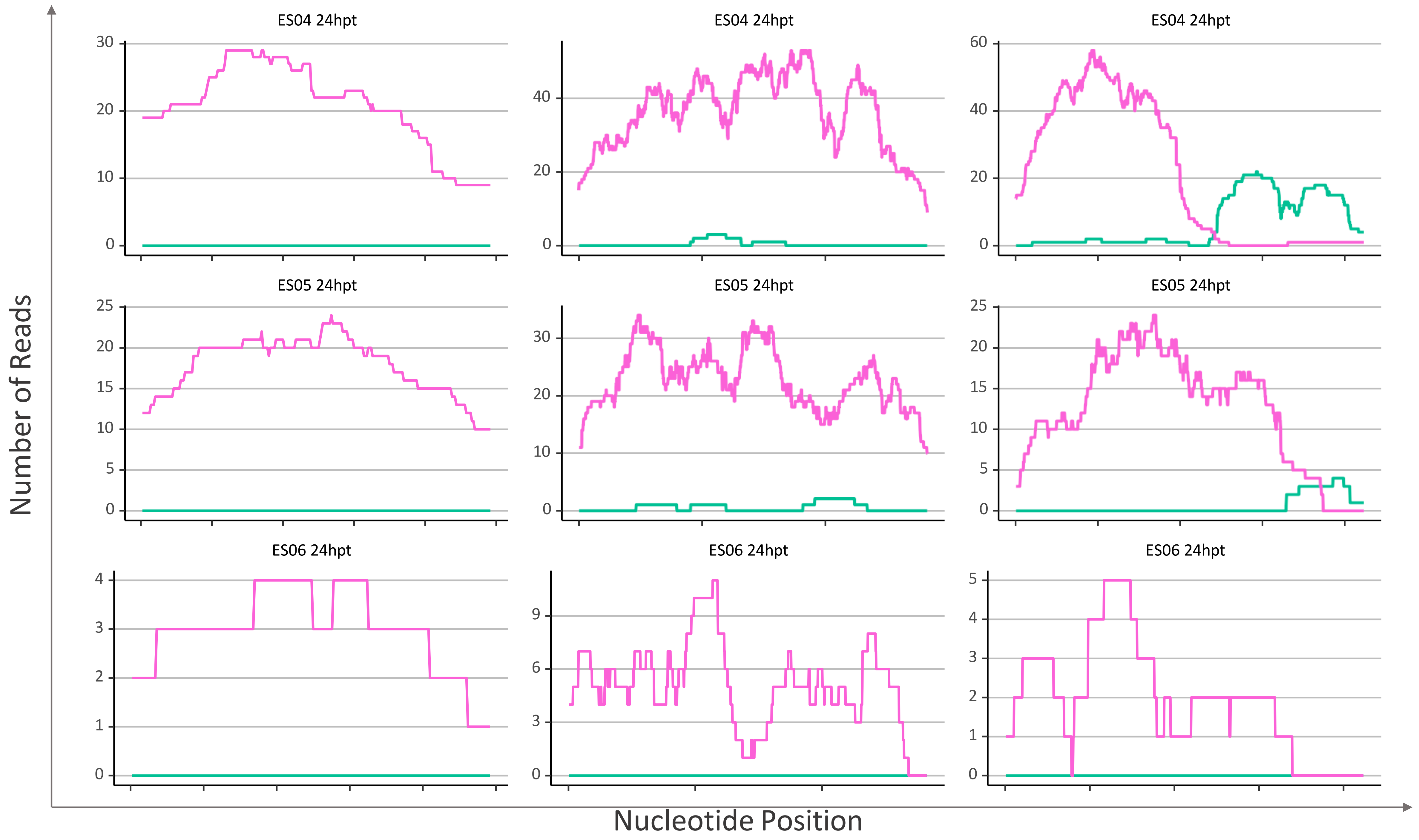

Number of Reads
Nucleotide Position
ES04 24hpt
ES04 24hpt
ES04 24hpt
ES05 24hpt
ES05 24hpt
ES05 24hpt
ES06 24hpt
ES06 24hpt
ES06 24hpt
0
10
20
30
0
20
40
0
20
40
60
0
5
10
15
20
25
0
10
20
30
0
1
2
3
4
0
3
6
9
0
1
2
3
4
5

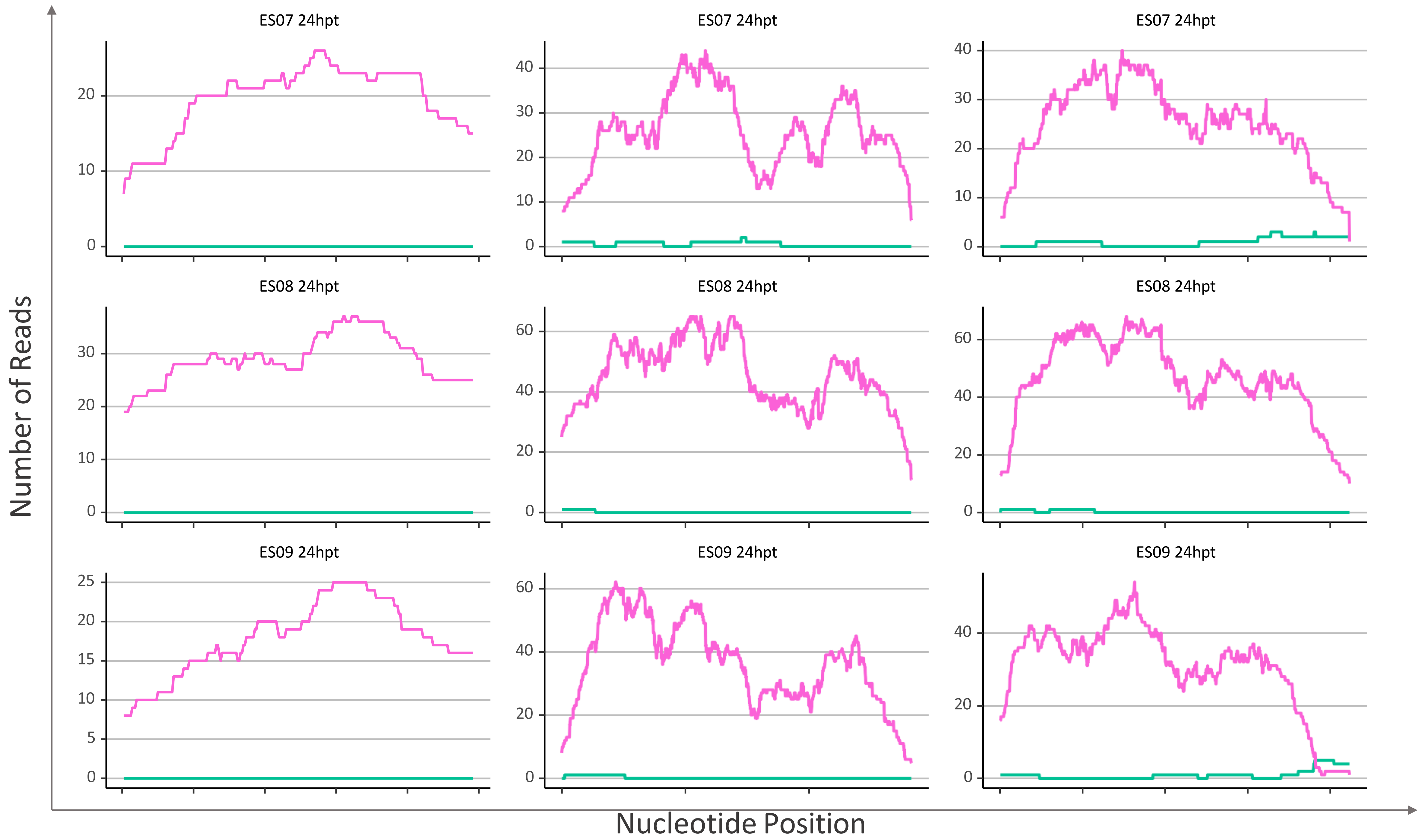

Number of Reads
Nucleotide Position
ES07 24hpt
ES07 24hpt
ES07 24hpt
ES08 24hpt
ES08 24hpt
ES08 24hpt
ES09 24hpt
ES09 24hpt
ES09 24hpt

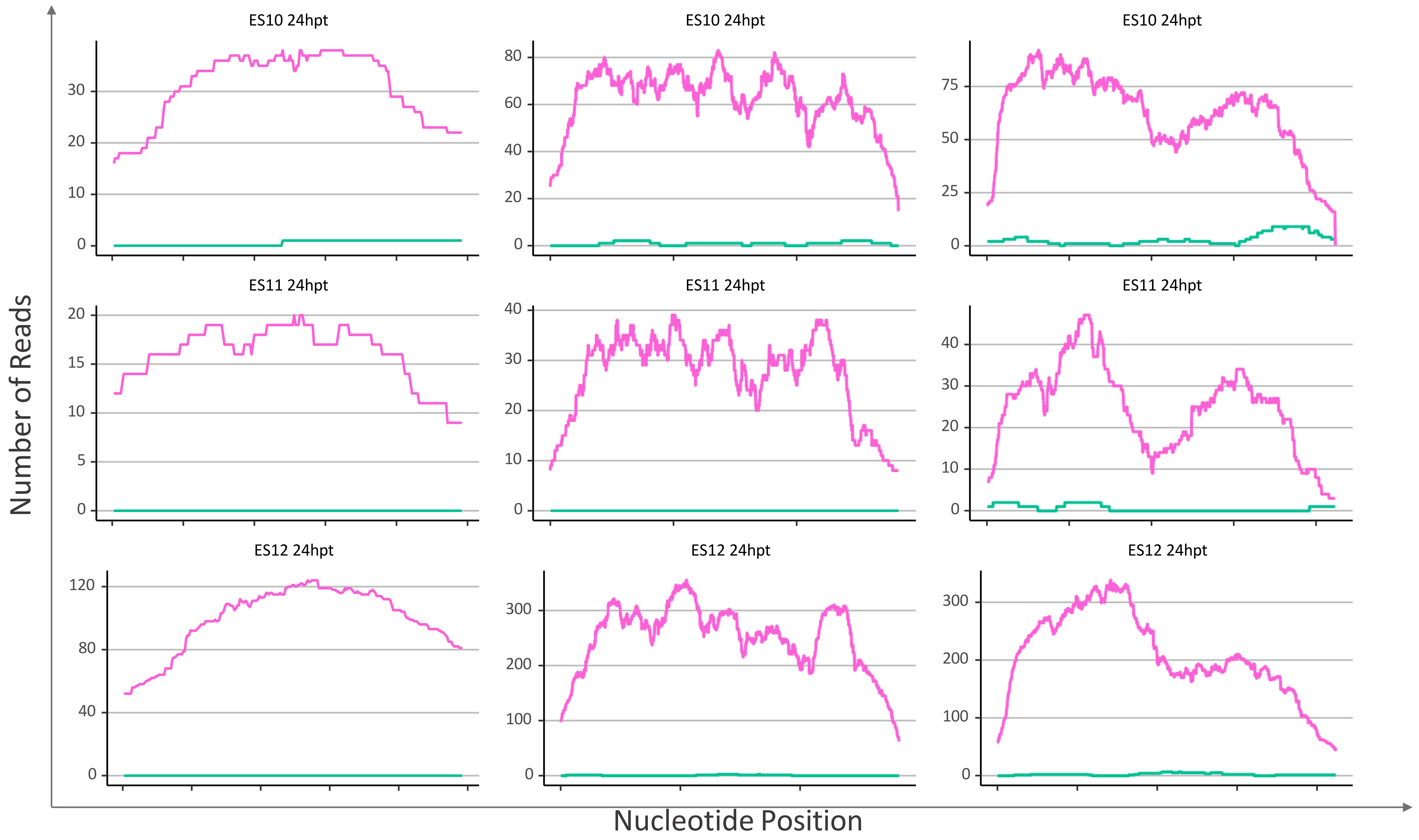

Number of Reads
Nucleotide Position
ES10 24hpt
ES10 24hpt
ES10 24hpt
ES11 24hpt
ES11 24hpt
ES11 24hpt
ES12 24hpt
ES12 24hpt
ES12 24hpt

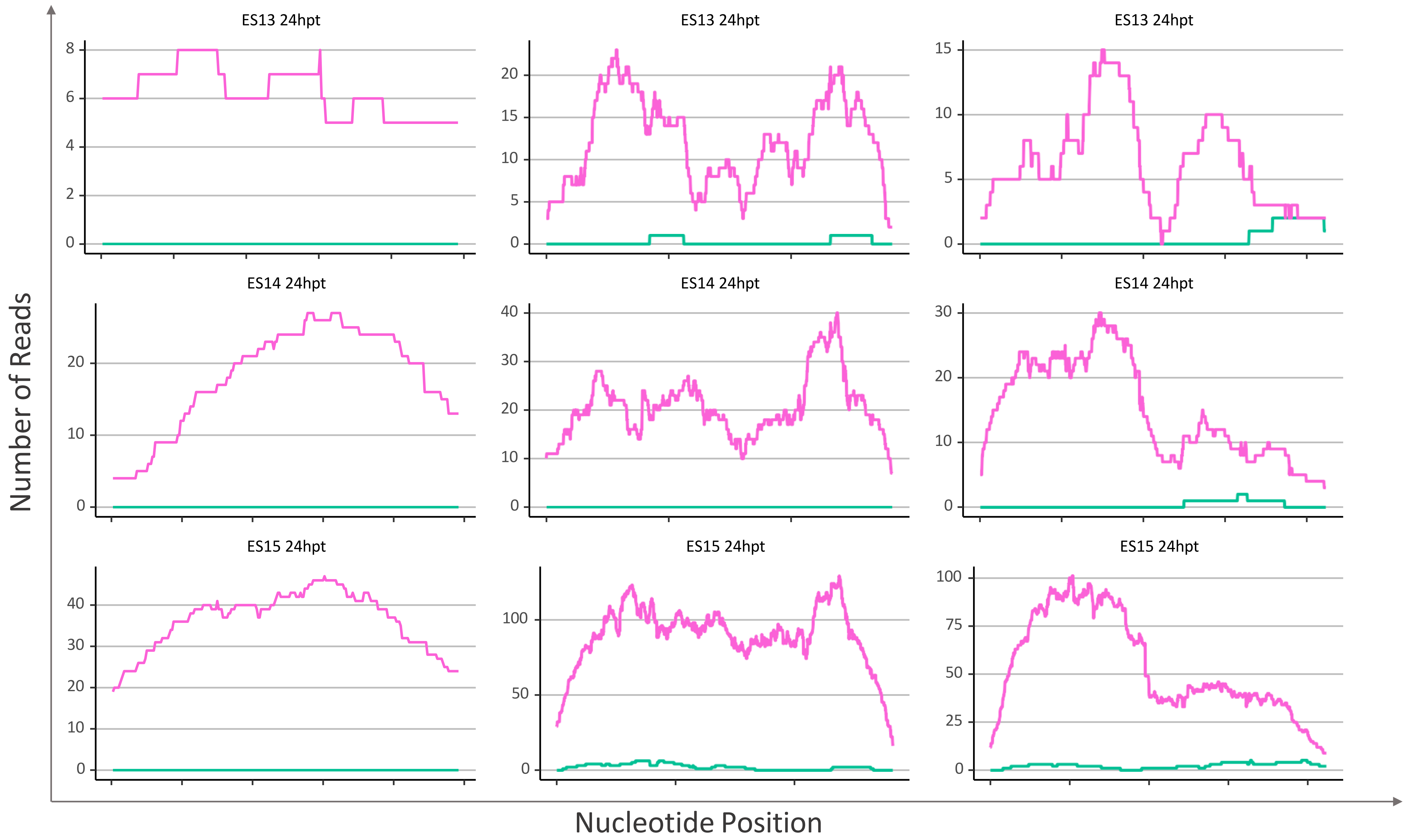

ES13 24hpt
ES13 24hpt
ES13 24hpt
ES14 24hpt
ES14 24hpt
ES14 24hpt
ES15 24hpt
ES15 24hpt
ES15 24hpt
Number of Reads
Nucleotide Position

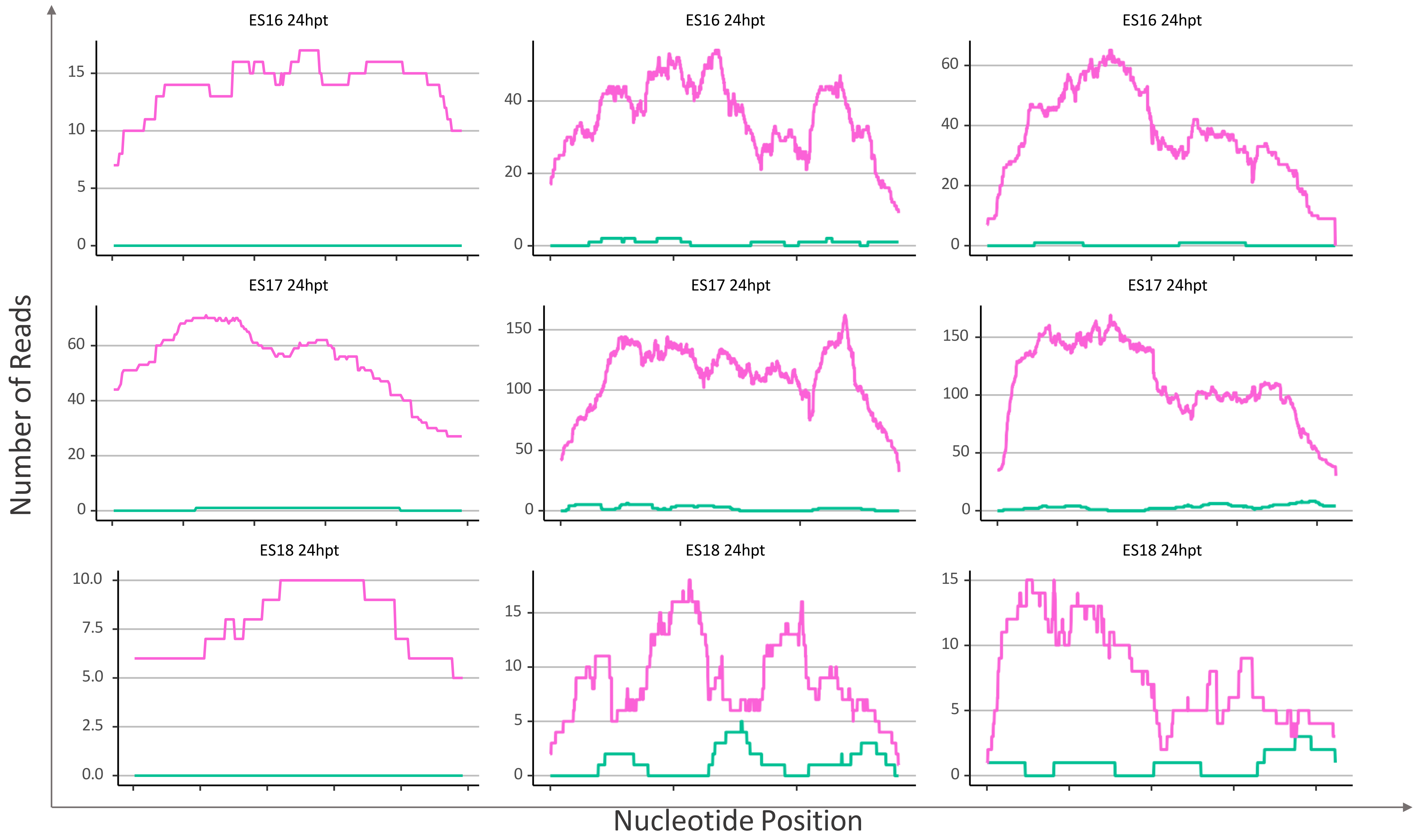
ES16 24hpt
ES16 24hpt
ES16 24hpt
ES17 24hpt
ES17 24hpt
ES17 24hpt
ES18 24hpt
ES18 24hpt
ES18 24hpt
Number of Reads
Nucleotide Position

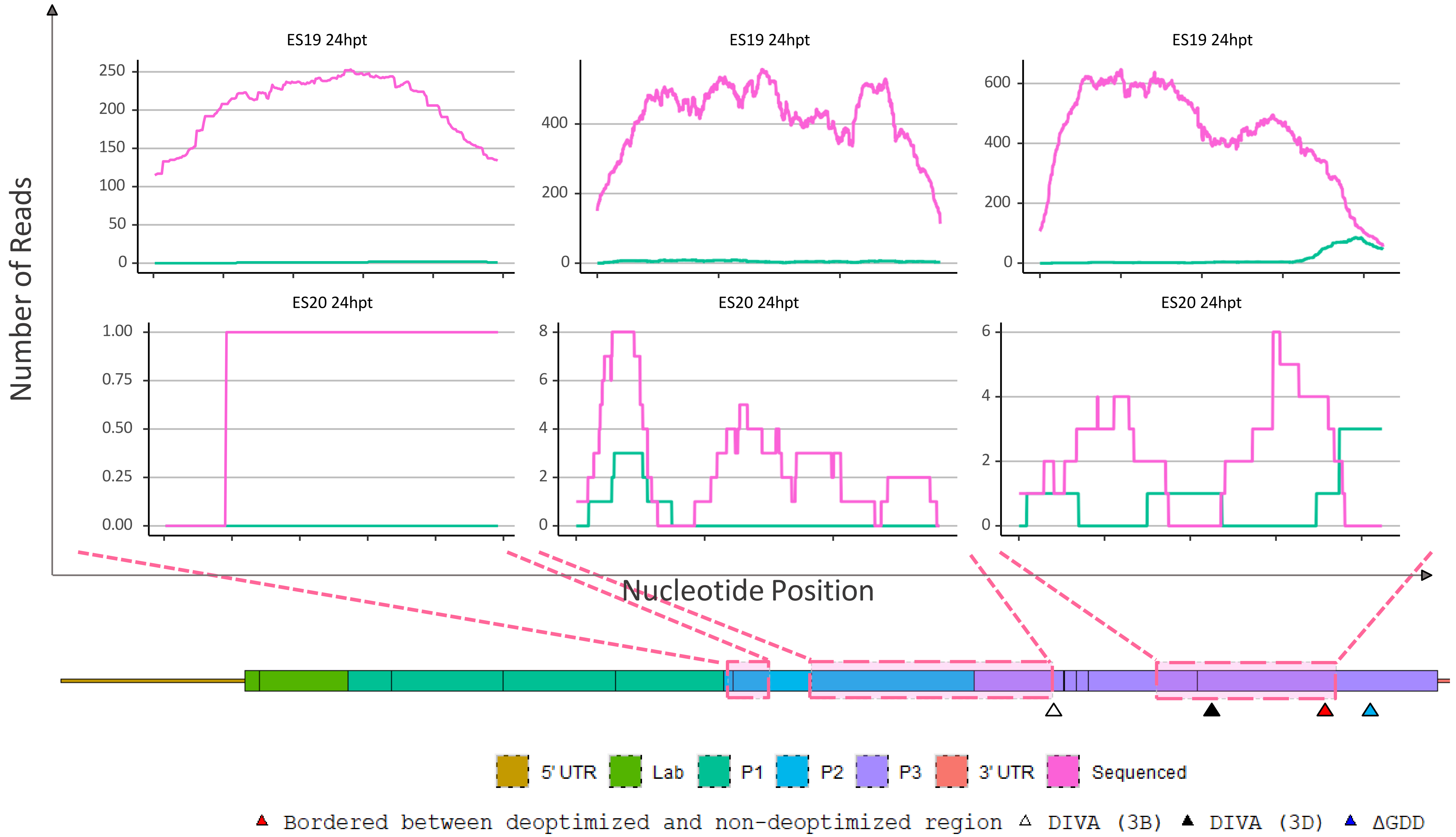

Number of Reads
ES19 24hpt
ES19 24hpt
ES19 24hpt
ES20 24hpt
ES20 24hpt
ES20 24hpt
Nucleotide Position
5' UTR
Lab
P1
P2
P3
3' UTR
Sequenced
Bordered between deoptimized and non-deoptimized region
DIVA (3B)
DIVA (3D)
ΔGDD
